# Flare, Persistently Active Disease, and Serologically Active Clinically Quiescent Disease in Systemic Lupus Erythematosus: A 2-Year Follow-Up Study

**DOI:** 10.1371/journal.pone.0045934

**Published:** 2012-09-21

**Authors:** Fabrizio Conti, Fulvia Ceccarelli, Carlo Perricone, Francesca Miranda, Simona Truglia, Laura Massaro, Viviana Antonella Pacucci, Virginia Conti, Izabella Bartosiewicz, Francesca Romana Spinelli, Cristiano Alessandri, Guido Valesini

**Affiliations:** Lupus Clinic, Reumatologia, Dipartimento di Medicina Interna e Specialità Mediche, Sapienza Università di Roma, Rome, Italy; University of Patras Medical School, Greece

## Abstract

**Objective:**

Several indices have been proposed to assess disease activity in patients with Systemic Lupus Erythematosus (SLE). Recent studies have showed a prevalence of flare between 28–35.3%, persistently active disease (PAD) between 46%–52% and serologically active clinically quiescent (SACQ) disease ranging from 6 to 15%. Our goal was to evaluate the flare, PAD and SACQ rate incidence in a cohort of SLE patients over a 2-year follow-up.

**Methods:**

We evaluated 394 SLE patients. Flare was defined as an increase in SLEDAI-2K score of ≥4 from the previous visit; PAD was defined as a SLEDAI-2K score of ≥4, on >2 consecutive visits; SACQ was defined as at least a 2-year period without clinical activity and with persistent serologic activity.

**Results:**

Among the 95 patients eligible for the analysis in 2009, 7 (7.3%) had ≥1 flare episode, whereas 9 (9.4%) had PAD. Similarly, among the 118 patients selected for the analysis in 2010, 6 (5%) had ≥1 flare episode, whereas 16 (13.5%) had PAD. Only 1/45 patient (2.2%) showed SACQ during the follow-up.

**Conclusion:**

We showed a low incidence of flare, PAD and SACQ in Italian SLE patients compared with previous studies which could be partly explained by ethnic differences.

## Introduction

Monitoring of disease activity is an important aspect in the management of patients affected by Systemic Lupus Erythematosus (SLE) as was recently pointed out in a core-set of recommendations proposed by the European League Against Rheumatism (EULAR) [1]. In clinical practice and in randomized controlled trials, several validated disease activity indices, derived from cohort or cross-sectional studies, have been widely applied [2], [3]. The EULAR recommendations for monitoring patients with SLE suggest that at least one validated index should be used to assess disease activity at each visit [1].

Flare is one of the most commonly used outcome measures in the core-set of indices evaluated in clinical trials on SLE. By using the existing disease activity indices, several definitions of flare have been proposed. Thus, a critical question is how to best define SLE flare. One of the most used was proposed by Gladman and colleagues in 2000 [3]. They defined flare when the SLE disease activity index (SLEDAI) score increases 4 or more points from the previous visit [3]. The investigators of the “Safety of Estrogen in Lupus National Assessment” (SELENA) group introduced a distinction between “mild/moderate” and “severe” flare. The authors emphasized that such distinction could be made on the basis of the *intention to treat* the flare [4].

More recently, Nikpour and colleagues underlined that such definition of flare does not capture patients who have a disease course characterized by periods of persistently active disease (PAD), defined as a SLEDAI-2K score ≥4, excluding serology alone, on ≥2 consecutive visits [5]. The authors observed that periods of PAD were more common than flare episodes, a result that we further confirmed in a subsequent evaluation on an Italian SLE population [5], [6].

“Serologically active clinically quiescent” (SACQ) disease was proposed as another outcome measure. This index identifies patients clinically quiescent despite persistent serologic activity, and appears to have a prevalence of 6–15% in SLE patients [7]–[9].

Thus, our goal was to evaluate the incidence of flare, PAD, and SACQ in a cohort of Italian SLE patients over a two-year follow-up.

## Materials and Methods

SLE patients referred to the Lupus Clinic of the Rheumatology Unit, Sapienza University of Rome (Sapienza Lupus Cohort) were enrolled in a prospective study. SLE diagnosis was performed according to the revised 1997 American College of Rheumatology (ACR) criteria [10]. Two-hundred ninety four consecutive SLE patients were evaluated during a two-year follow-up (2009–2010). Patients provided a written informed consent at the time of the first visit. The local ethical committee of “*Policlinico Umberto I*”, Rome, approved the study. At each visit, the patients underwent a complete physical examination, the clinical and laboratory data were collected in a standardized, computerized, and electronically-filled form, which included demographics, past medical history with date of diagnosis, co-morbidities, previous and concomitant treatments.

Disease activity was assessed with the SLEDAI-2K, while chronic damage was measured with the Systemic Lupus International Collaborating Clinics/ACR Damage Index score (SLICC) [11]–[13]. All the patients were observed at least twice per year, even though most of the patients were evaluated quarterly. Selected patients could be followed more often, according to the clinical condition.

Prospectively collected data were used to determine the incidence of flares and PAD in 2009 and 2010. According to Nikpour and colleagues, flare was considered as an increase in SLEDAI-2K score of ≥4 from the previous visit with a minimum interval of 2 months between visits; PAD as a SLEDAI-2K score ≥4, excluding serology alone, on ≥2 consecutive visits, with a minimum interval of 2 months between visits [5]. The proportion of patients with ≥1 flare episode(s) or PAD period(s) was determined. We recorded the organ systems involvement at the time of the flare or during a period of PAD.

SACQ was defined as a period of at least two years without clinical activity but with persistent serologic activity (SLEDAI-2K score 2 or 4, due to positive anti-dsDNA antibody titers and/or hypocomplementemia, at each clinic visit).

### Therapy

The patients evaluated for flare and PAD did not have any restriction concerning medications given. The patients evaluated for SACQ were taking only anti-malarials, while those receiving corticosteroids or immunosuppressive medications were excluded [7].

### Statistical Evaluation

We used version 13.0 of the SPSS statistical package. Normally distributed variables were summarized using the mean ± SD, and non-normally distributed variables by the median and range. Wilcoxon’s matched pairs test and paired t-test were performed accordingly. Univariate comparisons between nominal variables were calculated using chi-square test or Fisher-test where appropriate. Two-tailed P values were reported, P values less than 0.05 were considered significant.

## Results

Ninety-five and one-hundred eighteen SLE patients were eligible for the study in 2009 and 2010, respectively, as they underwent at least two visits per year. The main clinical and laboratory parameters are reported in Table 1. Patients selected for inclusion in the analysis were mostly women (94.7% in 2009 and 94.9% in 2010). No statistically significant differences were found between patients evaluated in 2009 and 2010 regarding the mean age (39.7±12.6 years and 41.8±11.3 years, respectively) and mean disease duration (124.7±97.6 months and 134.8±92.4 months, respectively).

**Table 1:** Clinical, serological and therapeutical features of SLE patients.

*Characteristic*	*Patients evaluated in 2009 (N = 95)*	*Patients evaluated in 2010 (N = 118)*
M/F	5/90	6/112
Age (years) mean±SD	39.7±12.6	41.8±11.3
Disease duration (months) mean±SD	124.7±97.6	134.8±92.4
***Race***		
Caucasian (N/%)	93/97.9	115/97.4
Asian (N/%)	2/2.1	3/2.6
***Clinical Manifestations***		
Renal disorder N (%)	19 (20.0)	27 (22.8)
Serositis N (%)	4 (4.2)	3 (2.5)
Cytopenia N (%)	21 (22.1)	27 (22.9)
NPSLE N (%)	12 (12.6)	15 (12.7)
Muscoloskeletal N (%)	24 (25.2)	24 (20.3)
Mucocutaneous N (%)	28 (29.5)	29 (24.6)
***Immunological Manifestations***		
ANA N (%)	92 (96.8)	112 (95)
Anti-dsDNA N (%)	38 (40)	40 (34)
Low C3 N (%)	39 (41.0)	39 (33)
Low C4 N (%)	45 (47.4)	47 (39.8)
SLEDAI (mean±SD)	1.72±2.19	1.84±2.25
SLICC (mean±SD)	0.51±0.82	0.48±0.89
***Drugs***		
Hydroxychloroquine N (%)	62 (65.3)	67 (56.8)
Mycophenolate mofetil N(%)	22 (23.1)	27 (22.9)
Cyclophosphamide N(%)	1 (1)	1 (0.8)
Methotrexate N(%)	6 (6.3)	9 (7.6)
Cyclosporine A N(%)	5 (5.2)	9 (7.6)
Azathioprine N(%)	16 (16.8)	18 (15.2)

SD: Standard Deviation; NP: NeuroPsychiatric; ANA: Anti-Nuclear Antibody; anti-dsDNA: anti-double strand DNA; SLEDAI: Systemic Lupus Erythematosus Disease Activity Index; SLICC: Systemic Lupus International Collaborating Clinics.

* As stated in 1997 ACR Classification criteria for SLE.

The mean ± SD time interval between visits used to diagnose ﬂare and PAD in 2009 and 2010 was 3.6±0.6 months and 3.5±0.6 months, respectively.

Among the 95 patients selected for the analysis in 2009, 7 (7.3%) had 1 flare episode (only 1/7 showed 2 flare episodes), whereas 9 (9.4%) had PAD. Similarly, among the 118 patients selected for the analysis in 2010, 6 (5%) had 1 flare episode (none more than 1 episode), whereas 16 (13.5%) had PAD.

In 2009, 1 patient showed both flare and PAD. One patient had flare in 2009 and in 2010, while 5 patients showed PAD in both years.

Table 2 shows the clinical characteristics of patients with flare and the organ/system involved at the time of flare. In 2009, the most commonly involved organ/systems in patients with flare were immunologic and musculoskeletal (57.1% and 42.8%, respectively), while nervous system involvement was the most frequent manifestation in patients with flare in 2010 (66.6%). Specifically, 2 patients experienced psychosis, one patient organic brain syndrome, and one patient a new onset of cerebrovascular accident.

**Table 2:** Demographic characteristics of SLE patients with flare and the organ/systems involved at the time of flare.

Characteristic	*Patients with flare in 2009 (N = 7)*		*Patients with flare in 2010 (N = 6)*	
M/F	0/7		1/5	
Age (years) mean±SD	37.7±9.2		40.3±11.8	
Disease duration (months) mean±SD	187.2±115.2		188.4±100.08	
**Systemic involvement** *				
Renal disorder N(%)	1/14.3		0/0	
Serositis N(%)	0/0		1/16.6	
Cytopenia N(%)	0/0		1/16.6	
NPSLE N(%)	2/28.6		4/66.6	
Musculoskeletal N(%)	3/42.8		0/0	
Mucocutaneous N(%)	1/14.3		1/16.6	
Immunological abnormalities (besides ANA) N(%)	4/57.1		1/16.6	
Prednisone dosage (mg/week) mean±SD**	73.9±123.7	66.0±67.03		
**Drugs**				
Hydroxychloroquine N(%)	2/28.6	6/100		
Mycophenolate mofetil N(%)	2/28.6	0/0		
Cyclophosphamide N(%)	0/0	0/0		
Methotrexate N(%)	0/0	0/0		
Cyclosporine A N(%)	0/0	1/16.6		
Azathioprine N(%)	0/0	0/0		
SLEDAI (mean±SD)	6.8±3.02	8±1.26		
SLICC (mean±SD)	0.57±1.13	1.2±0.8		

SD: Standard Deviation; NP: NeuroPsychiatric; SLEDAI: Systemic Lupus Erythematosus Disease Activity Index; SLICC: Systemic Lupus International Collaborating Clinics.

* As stated in 1997 ACR Classification criteria for SLE.

** Prednisone equivalents.

Notably, 5/7 patients (71.4%) in 2009 and 5/6 (83.4%) in 2010 of the patients who experienced flares were not on immunosuppressive drugs. The patients who experienced flares showed a significantly longer disease duration compared with those who did not experience flares in both years of observation (187.2±115.2 *versus* 128.4±84.6 months, P = 0.02 in 2009; 188.4±100.08 *versus* 135.8±89.5 months, P = 0.03 in 2010).

The clinical characteristics of the patients with PAD and the involved organ/systems are reported in table 3. Musculoskeletal involvement and immunological abnormalities were found in 50% of the patients with PAD in 2009, while in 2010 kidney and nervous system involvement were the most frequent manifestations (37.5% and 25%, respectively). As seen in the group with flare, the patients with PAD showed a significantly longer disease duration compared with those who did not have PAD in both years of observation (184.8±118.32 *versus* 122.6±88.6 months, P = 0.02 in 2009; 188.4±100.08 *versus* 138.8±83.5 months, P = 0.02, in 2010).

**Table 3:** Demographic characteristics of SLE patients (N = 16) with PAD and organ/system involving during PAD.

Characteristic	*Patients with PAD in 2009 (N = 6)*	*Patients with PAD in 2010 (N = 16)*
M/F	1/5	1/15
Age (years) mean±SD	35.8±5.3	38.3±8.02
Disease duration (months) mean±SD	184.8±118.32	152.4±111.6
**Systemic involvement** *		
Renal disorder N(%)	2 (33.3)	6/37.5
Serositis N(%)	1/16.6	1/6.25
Cytopenia N(%)	1/16.6	2/12.5
NPSLE N(%)	1/16.6	4/25
Musculoskeletal N(%)	3/50	3/18.75
Mucocutaneous N(%)	0/0	2/12.5
Immunological abnormalities (besides ANA) N(%)	3/50	3/18.75
Prednisone dosage (mg/week) mean±SD**	38.3±6.05	67.3±86.3
**Drugs**		
Hydroxychloroquine N(%)	3/50	5/31.25
Mycophenolate mofetil N(%)	3/50	5/31.25
Cyclophosphamide N(%)	0/0	0/0
Methotrexate N(%)	2/33.3	2/12.5
Cyclosporine A N(%)	0/0	0/0
Azathioprine N(%)	1/16.6	3/18.75
SLEDAI (mean±SD)	5.8±1.86	5.96±1.94
SLICC (mean±SD)	0.33±0.56	0.71±1.437

SD: Standard Deviation; NP: NeuroPsychiatric; SLEDAI: Systemic Lupus Erythematosus Disease Activity Index; ECLAM: European Consensus Lupus Activity Measurement; SLICC: Systemic Lupus International Collaborating Clinics.

* As stated in 1997 ACR Classification criteria for SLE.

** Prednisone equivalents.

The occurrence of flare was associated with a history of nervous system involvement (P = 0.001, OR = 10.9, CI 2.1–56.8). The logistic regression analysis confirmed such association (P = 0.008). The occurrence of PAD was associated with a history of arthritis, renal and nervous system involvement (P = 0.007, OR = 5.7, CI 1.4–22.9; P<0.001, OR = 9.37, CI 2.6–33.7; P = 0.002, OR = 7.49, CI 1.7–32, respectively). The logistic regression analysis confirmed the association only with nervous system involvement (P = 0.01).

Forty-five patients were eligible for the evaluation of SACQ. Only 1 patient (2.2%) had SACQ during the two-year follow-up. The patient (D.G.) was a 37-year-old female, with a disease duration of 144 months, who was taking hydroxychloroquine and, in the absence of clinical manifestations, showed persistent complement reduction and elevated anti-dsDNA antibodies (titer ≥1∶40).

## Discussion

In this prospective study, we showed that, in a large cohort of Italian patients affected by SLE, flares and PAD are relatively infrequent conditions. In fact, in 2009 flares were observed in 7% and PAD in 9.4% of our patients, while in 2010 flares were observed in 5% and PAD in 13.5% of our patients, respectively.

SLE is a prototype of systemic autoimmune diseases, characterized by heterogeneity of clinical features and presence of a wide autoantibodies profile. The great heterogeneity of SLE determined a still open debate, concerning the possibility that SLE is a single disease with varied phenotypes or a similar phenotype shared by a variety of different diseases with diverse pathogenic mechanisms [14].

SLE is characterized from heterogeneous degrees of severity as well as unpredictable disease flares and remissions. Several experimental trials are in progress to evaluate new biologic drugs to treat patients affected by SLE. However, the remitting and relapsing course of the disease and the heterogeneity of SLE features make the design and the interpretation of clinical trials difficult. Quantification of disease activity is mandatory to identify patients eligible to participate in clinical trials and, thereafter, to establish the efficacy of the drug examined.

Data published in the literature suggested that the disease activity in SLE patients could be evaluated by using laboratory markers and/or global indices. Conventional biomarkers for the assessment of disease activity include anti-dsDNA antibodies and serum complement levels. However, the “classical” markers of activity are not specific and accurate in differentiating between disease flares and other concomitant conditions, such as infections. Recent research has provided data about new potential biomarkers to guide clinical decision-making in the management of SLE patients [15].

Disease activity indices are helpful in the routine assessment of SLE patients, as suggested by recent EULAR recommendations for monitoring patients with SLE [1].

Several indices have been applied to evaluate disease activity, such as SLEDAI, European Consensus Lupus Activity Measurement (ECLAM) and British Isles Lupus Assessment Group index (BILAG) [16].

Recent randomized controlled trials for new biologic treatment in patients affected by SLE used a new composite assessment, called SLE Responder Index (SRI), that combines the SELENA-SLEDAI, BILAG and Physician Global Assessment (PGA). A responder according to the SRI is defined as having ≥4 point reduction from baseline in SELENA-SLEDAI score and no new BILAG A score and no more than one new BILAG B organ domain score compared with baseline and no worsening in PGA. When all three criteria are met, the patient is a responder at that time point according to the SRI [17].

Several definitions of flare were proposed and used in clinical trials and observational studies. Nevertheless, how to define the best SLE flare is still an open and critical question. Global scoring systems, such as the SLEDAI, are easy to perform and widely standardized. They are used to define flare; nonetheless they only take a snapshot of the patient rather than indicating the trend of the disease.

Thus, Nikpour et al. proposed a new outcome measure, the so-called “persistently active disease” (PAD), to define those patients not in remission but without flare. They addressed that these patients require the most frequent monitoring [5]. Their study published in 2009 showed a high prevalence of flare and PAD in a Canadian lupus cohort. At least 1 flare was registered in nearly one third of evaluated patients (specifically 35.3% in 2004 and 28% in 2005), while PAD was identified in almost half of the patients evaluated during the two-year follow-up (specifically, 52.3% in 2004 and 46.1% in 2005). The most commonly involved organ/systems were musculoskeletal, cutaneous, renal, immunologic, and the nervous system [5].

We have already reported a preliminary study on 63 SLE patients who were referred to the Sapienza Lupus Clinic followed during 1 year of follow-up (September 2008–September 2009) in which we observed a lower incidence of flare and PAD compared with the Canadian cohort (7.9% and 14.3%, respectively) [6]. In the present study, we extended the time and cohort size the preliminary data on Italian SLE patients. This two-year follow-up study confirmed a lower incidence of flare (7% in 2009 and 5% in 2010), and PAD (9.4% in 2009 and 13.5% in 2010), indicating a relatively infrequent occurrence of relapses and a good control of disease activity. Ethnic differences, i.e., the presence of one-third of African-American patients in the Canadian cohort, could explain the better outcome of our patients. Nonetheless, we observed a more frequent involvement of the central nervous system and less frequent of the skin during flares or PAD. Neuropsychiatric involvement is frequent manifestations in SLE patients and could be found up to 80% of patients, includeing a wide range of neurological and psychiatric manifestations as well as cognitive impairment. Recently we found an association between cognitive dysfunction and disease activity in a cohort of 58 consecutive SLE patients [18].

Occurrence of flares and PAD in our SLE cohort was associated with longer disease duration. Moreover, most patients who experienced flares were not taking an immunosuppressive drug. Thus, longer disease duration and the absence of an immunosuppressive treatment should be considered risk factors for the worsening of disease activity.

In the logistic regression analysis NPSLE involvement was associated with both flare and PAD. This is in agreement with the definition of flare, as well as of PAD, probably due to the fact that in the SLEDAI, used in the formulation of both indices, neuropsychiatric manifestations account for the highest scores.

SACQ is another important outcome measure that was first suggested by Gladman et al. in 1979. They described a subset of patients who had persistent serologic activity (elevated anti-dsDNA antibody levels and/or hypocomplementemia) despite clinical quiescence [8]. Walz & LeBlanc reported 12% of SLE patients with SACQ [15], and even a lower percentage was found (6.1%) by Steiman and colleagues [7] who further found that 58.9% of patients with SACQ may experience flare at median 155 weeks of follow-up. Changes in complement and anti-dsDNA antibody serum levels drawn at routine clinic visits might not be predictive of flares in SACQ patients. Thus, it was suggested that the decision to treat patients with SACQ should be based on close clinical observation. In our population, only 1 (2.2%) of the 45 eligible patients showed SACQ. We identified a lower incidence of SACQ in SLE patients, but the importance of this index in the clinical assessment should be addressed in larger cohorts.

### Conclusions

Our study showed a low incidence of flares, PAD and SACQ in Italian SLE patients compared with previous studies where the results could be only partly explained by ethnic differences. This may suggest that definition of disease activity is critical for SLE management, and that timing for immunosuppressive treatment suspension should be carefully evaluated. In this view, it is very important to improve, select and use indices of outcome in SLE in order to better assess and treat patients. Flares, PAD and SACQ could be considered useful parameters of clinical evaluation in SLE patients in monitoring disease progression and response to treatment.
